# Nanomedicine-based adjuvant therapy: a promising solution for lung cancer

**DOI:** 10.1186/s12951-023-01958-4

**Published:** 2023-07-06

**Authors:** Yiming Xu, Jessica C. Hsu, Liyun Xu, Weiyu Chen, Weibo Cai, Kai Wang

**Affiliations:** 1 Department of Respiratory Medicine, The Fourth Affiliated Hospital, Zhejiang University School of Medicine, Yiwu, 322000 Zhejiang China; 2 Departments of Radiology and Medical Physics, University of Wisconsin-Madison, Madison, WI 53705 USA; 3 International Institutes of Medicine, The Fourth Affiliated Hospital of Zhejiang University School of Medicine, Yiwu, Zhejiang China

**Keywords:** Nanomedicine, Lung cancer, Adjuvant therapy, Theranostics

## Abstract

Lung cancer has been the leading cause of cancer-related deaths worldwide for decades. Despite the increasing understanding of the underlying disease mechanisms, the prognosis still remains poor for many patients. Novel adjuvant therapies have emerged as a promising treatment method to augment conventional methods and boost the therapeutic effects of primary therapies. Adjuvant therapy based on nanomedicine has gained considerable interest for supporting and enhancing traditional therapies, such as chemotherapy, immunotherapy, and radiotherapy, due to the tunable physicochemical features and ease of synthetic design of nanomaterials. In addition, nanomedicine can provide protective effects against other therapies by reducing adverse side effects through precise disease targeting. Therefore, nanomedicine-based adjuvant therapies have been extensively employed in a wide range of preclinical and clinical cancer treatments to overcome the drawbacks of conventional therapies. In this review, we mainly discuss the recent advances in adjuvant nanomedicine for lung cancer treatment and highlight their functions in improving the therapeutic outcome of other therapies, which may inspire new ideas for advanced lung cancer therapies and stimulate research efforts around this topic.

**Graphical Abstract:**

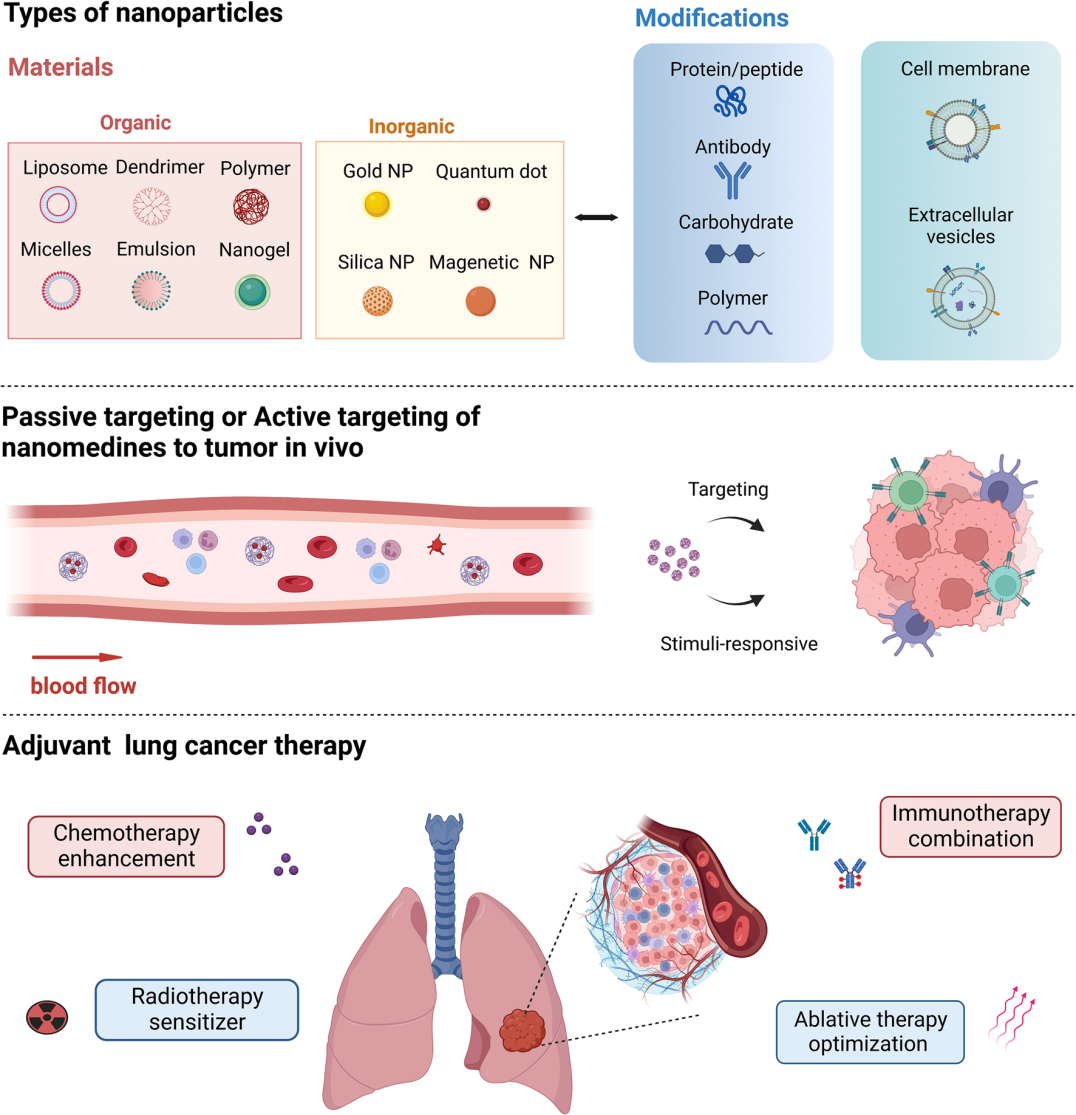

## Introduction

Lung cancer has wrought negative impact upon public health worldwide. Although research around lung cancer has experienced explosive growth over the last two decades, the morbidity and mortality rates of lung cancer have remained high [1]. Nearly 2.2 million people are diagnosed with lung cancer and 1.8 million people die from this disease yearly [2]. Thus, there is an urgent need to develop alternative therapeutic approaches to prevent the progression of lung cancer and improve long-term survival.

Generally, lung cancer can be divided into two subtypes, including non-small cell lung cancer (NSCLC) and small cell lung cancer (SCLC). NSCLC and SCLC account for approximately 85% and 15% of all lung cancer cases, respectively [3]. For early-stage NSCLC, surgical resection is the most effective therapy, while adjuvant chemotherapy improves the survival in patients with stage II and IIIA NSCLC [4]. For locally advanced, unresectable NSCLC, the standard-of-care comprises a combination of chemotherapy and radiotherapy [4]. Molecularly targeted therapy and immunotherapy have also provided great advantages in managing NSCLC [4, 5]. As for SCLC, which is characterized by late diagnosis and poor prognosis, traditional chemotherapy is commonly given as first-line treatment, while radiotherapy is usually employed for patients with limited-stage disease [6]. In addition, targeted therapy, immunotherapy, and other therapy combinations have been continually explored and evaluated for SCLC treatment with promising but varying results [6, 7]. However, traditional therapies are often hampered by many obstacles, such as low efficiency, drug resistance, and inevitable side effects, which significantly diminish their therapeutic efficacy.

Nanomedicine has shown great potential in cancer screening and diagnosis, drug delivery, and therapy enhancement [8]. It is recognized that nanomaterials can accumulate inside tumors via the enhanced permeability and retention (EPR) effect, thus reducing systemic side effects [9]. However, the delivery efficiency is still not satisfactory. Therefore, a series of strategies have been developed to enhance the delivery of nanomedicines, such as active tumor-targeting via surface ligands or bio-mimetic designs, tumor priminig via pharmacological and physical co-treatments, and use of multi-stage or stimuli-responsive nanocarrier materials [10]. Indeed, advancement has been seen in the field of nanomedicine with the recent development of cancer nanotheranostics, especially for lung cancer [11]. Several nano-based formulations are undergoing clinical investigations as adjuvant therapies for lung cancer. This review will highlight the progress of adjuvant nanomedicine for lung cancer treatment and discuss the emerging nanomedicine-based therapeutic approaches in details.

## Current adjuvant therapies in the clinic

Cancer adjuvant therapies usually refer to the additional treatment performed after tumor resection surgery, with the main purpose of eliminating residual micro-cancer cells to reduce the risk of recurrence caused by residual cancer cells. Current clinical adjuvant treatments for lung cancer include chemotherapy, radiotherapy, molecularly targeted therapy, immunotherapy, and ablative therapies based on radiofrequency and light. Historically, chemotherapy and radiotherapy are the major adjuvant treatment options for lung cancer. Cisplatin-based doublet (preferentially cisplatin plus vinorelbine) is generally applied to stage II or III NSCLC patients with completely resected tumors [12]. In addition, neo-adjuvant chemotherapy has shown to improve survival rate of early-stage NSCLC in some clinical studies [13]. However, due to the challenges associated with delayed surgery, there is no preferred standard regimen for neo-adjuvant chemotherapy. Furthermore, the benefit of adjuvant radiotherapy for stages I–III NSCLC patients undergoing surgical resection is still controversial, though it is considered a standard treatment for those with positive surgical margins [14]. For patients with limited-stage SCLC, cisplatin plus etoposide in combination with thoracic radiotherapy is given as first-line therapy. Lastly, patients with local NSCLC, who are not suited for surgical resection, usually receive alternative treatments, such as radiofrequency ablation (RFA) therapy, photodynamic therapy (PDT), and photothermal therapy (PTT) [15, 16].

The recent advances in high-throughput sequencing technologies, particularly next-generation sequencing (NGS), have enabled the molecular characterization of NSCLC. This has led to the identification of a series of oncogenic alterations that may serve as potential targets for drug development [17]. To date, several therapies targeting tyrosine kinase inhibitors (TKIs) have been established as first-line treatment in the clinic, such as TKI rearrangements for epidermal growth factor receptor (EGFR) exon 19 deletion and L858R mutations, ALK and ROS1 [5]. In addition, rearrangements for other targets such as Kirsten rats arcomaviral oncogene homolog (KRAS) G12C mutations, mesenchymal epithelial transition factor (MET) exon 14 alterations, NeuroTrophin Receptor Kinase (NTRK) and ret proto-oncogene (RET) are rapidly developing. Furthermore, immunotherapy has become a promising strategy for managing lung cancer. Immune checkpoint inhibitors (ICIs) have been widely utilized as first-line therapy, either as single agents or in combination with chemotherapy [12]. First-line chemotherapy in conjunction with ICIs can significantly improve progression-free survival as seen in a number of clinical trials. The combined therapy has demonstrated better overall survival for NSCLC and extensive-stage SCLC patients than that of a single treatment alone [18, 19]. In addition, reinforcement using ipilimumab (CTLA-4 blockade) can induce inhibitory effects on chemo-refractory metastatic NSCLC after palliative radiation [20]. Other than ICIs, many non-specific immunotherapies, such as those based on cytokines, Toll-like receptors (TLR) agonists, and cancer vaccines, are currently under evaluation [21].

## Therapies enhanced by adjuvant nanomedicine

### Chemotherapy enhancement

Conventional systemic chemotherapy is the backbone of lung cancer treatment. However, the benefits of chemotherapy can be limited due to issues such as poor aqueous solubility, non-specific cellular toxicity, low drug efficiency and adverse side effects. For example, platinum-based compounds, such as cisplatin and carboplatin, are the most commonly used chemotherapy drugs, but they often induce unwanted off-target effects, such as peripheral neuropathy, nephrotoxicity, and myelosuppression [22]. On the other hand, functionalized nanomaterials have the potential to improve therapeutic efficacy by minimizing non-specific cellular toxicity via bio-responsive reaction, enabling targeted delivery, and increasing circulation time via PEGylation. To date, two nano-based chemotherapeutics for lung cancer have been clinically approved, while multiple formulations are currently undergoing clinical trials (Table 1). Abraxane (Nab-paclitaxel), an FDA-approved solvent-free nanomedicine, is a human serum albumin-bound paclitaxel (PTX) nano-formulation with a size of 130 nm [23]. Abraxane can dissolve into soluble albumin-PTX complexes upon injection, although some PTX may bind to other biomolecules or exist as PTX only [24]. Notably, Nab-paclitaxel has demonstrated better therapeutic efficacy and more favorable drug distribution and delivery than traditional solvent-based(sb)-PTX. As identified in a phase III clinical trial study, Nab-paclitaxel is considered as a standard therapy for advanced NSCLC patients who have previously undergone treatments [25]. Nab-paclitaxel alone (NCT04213937) or in combination with gemcitabine (NCT02769832) is currently being investigated in phase II clinical trials as a treatment regimen for advanced SCLC. Genexol-PM, another FDA-approved nanoformulation of PTX, is a polymeric micelle (diameter ~ 23.91 nm) that employs monomethoxy polyethylene glycol-block-poly(D,L-lactide) (mPEG-PDLLA) to encapsulate PTX (16 wt%) [26]. Genexol-PM can also rapidly dissolve into soluble albumin-bound PTX complexes after intravenously administration and have shown better pharmacokinetics than Nab-paclitaxel due to the PEG surface layer. Other lung cancer specific nano-chemotherapeutics based on cisplatin, docetaxel, camptothecin, and irinotecan are currently undergoing clinical investigations (Table 1) [27].

**Table 1:** Adjuvant nanomedicine approved or undergoing clinical trials for lung cancer therapy

Nanomedicine	Formulation	Combination	Application	Phase of study	Current status	Institution	ClinicalTrials.gov Identifier	
Abraxane (Nab-paclitaxel)	Albumin-bound PTX	/	NSCLC	FDA approved	/	Abraxis BioScience	/	
Genexol-PM	PTX-loaded micelle (PEG-PLGA)	/	NSCLC	Approved in Korea	/	Samyang Holdings Biopharmaceuticals	/	
Lipusu	PTX liposome	Gemcitabine; Cisplatin	Advanced Squamous NSCLC	Phase IV	Unknown	Nanjing Luye Sike Pharmaceutical Co.	NCT02996214	
CT-2103 (Xyotax)	Polyglutamate PTX	Pemetrexed	Advanced NSCLC	Phase II	Completed	Dartmouth-Hitchcock Medical Center	NCT00487669	
NC-6004	Nanoparticle cisplatin	Gemcitabine	NSCLC	Phase I/II	Completed	NanoCarrier Co.	NCT02240238	
Doxil	Pegylated liposomal doxorubicin	Topotecan	SCLC	Phase I	Completed	Christiana Care Health Services	NCT00252889	
		ATRC-101; Pembrolizumab	NSCLC	Phase I	Recruiting	Atreca, Inc.	NCT04244552	
BIND-014	Docetaxel nanoparticles	/	NSCLC	Phase II	Completed	BIND Therapeutics	NCT01792479	
		/	KRAS Positive or Squamous NSCLC	Phase II	Completed	BIND Therapeutics	NCT02283320	
CRLX101 (NLG207)	Camptothecin conjugated to a cyclodextrin-based polymer	/	Advanced NSCLC	Phase II	Completed	Lumos Pharma (NewLink Genetics Corporation)	NCT01380769	
NKTR-102	Pegylated irinotecan	/	Relapsed SCLC	Phase II	Completed	Roswell Park Cancer Institute	NCT01876446	
		/	Metastatic and Recurrent NSCLC	Phase II	Completed	Abramson Cancer Center of the University of Pennsylvania	NCT01773109	
Onivyde	Irinotecan liposome	Topotecan	SCLC	Phase II/III	Active, not recruiting	Ipsen	NCT03088813	
Topotecan liposome	Topotecan liposome	/	Advanced SCLC	Phase I	Recruiting	Fujifilm Pharmaceuticals U.S.A., Inc.	NCT04047251	
		/	Advanced SCLC	Phase I	Completed	Spectrum Pharmacerticals, Inc	NCT00765973	
OSI-211 (NX-211)	Liposomal Lurtotecan	/	Recurrent SCLC	Phase II	Completed	Astellas Pharma Inc	NCT00046787	
		Cisplatin	Advanced or Metastatic lung cancer	Phase I	Completed	Canadian Cancer Trials Group	NCT00006036	

*PTX* paclitaxel, *FDA* Food and Drug Administration, *PEG* polyethylene glycol, *PLGA* polylactic-co-glycolic acid

An increasing number of studies have indicated the potential of nanomedicine in enhancing chemotherapeutic effects. For example, Sun et al. enhanced the therapeutic efficacy of PTX by using a multistage tumor-targeting liposome that contains two targeted peptide-modified lipids, including cRGD-PEG_2000_-DSPE and KLA-PEG_2000_-DSPE [28]. The results showed that the PTX liposomal nanoparticles exhibit strong tumor growth inhibition (80.6%) and antiangiogenic effects without inducing systemic toxicity in tumor-xenografted BALB/c mice [28]. The liposomal formulation, which contains cyclic derivatives of RGD (Arg-Gly-Asp) oligopeptides, can selectively bind to the α_v_β_3_ integrin that is highly expressed in tumor cells, such as lung cancer, breast cancer, and activated vascular endothelial cells. The formulation also includes another peptide, D-(KLAKLAK)2 (KLA), which is a positively-charged, mitochondria-targeting sequence that can target and disrupt the mitochondrial membrane.

In addition to synthetic lipids or peptides, natural bio-membrane (including cellular membranes and extracellular vesicles) coatings have shown great success in improving drug delivery in various tumor types including lung cancer [29]. For example, by using red blood cell membrane (RBCm) wrapping technology, Gao et al. constructed RBCm wrapped pH-sensitive poly(l-γ-glutamylcarbocistein)-PTX nanoparticles to prolong the blood circulation time and allow for timely release of PTX in acidic tumor microenvironment (TME) [30], and these RBCm wrapped nanoparticles exhibited significantly stronger antitumor effect (P < 0.001) in NSCLC tumor-bearing mice than non-wrapped nanoparticles. Moreover, Agrawal et al. prepared milk-derived exosomes and loaded them with PTX (Exo-PTX) [31]. Oral administration of Exo-PTX in nude mice bearing human lung carcinoma xenografts resulted in significant inhibition of tumor growth (60%) and remarkably lower systemic and immunologic toxicities as compared to intravenously injected PTX [31].

**Fig. 1:**
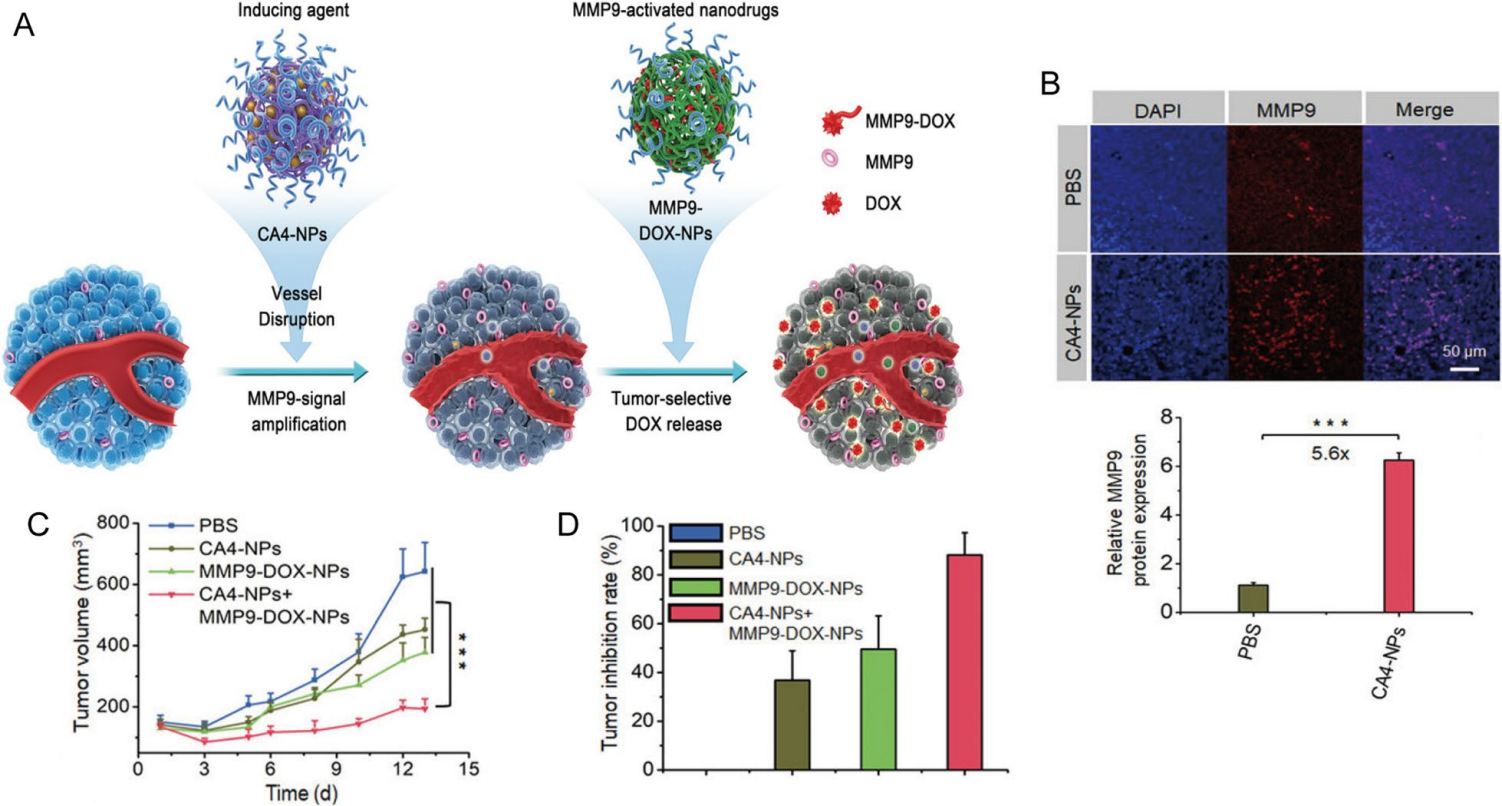
** A** Scheme illustration of cooperative cancer treatment by combining CA4 nanodrug and MMP9-activated DOX prodrug nanomedicine. **B** Immunofluorescence images and quantitative analysis of MMP9 in tumor tissues. **C** Tumor volume and **D** tumor inhibition rate of 4T1 tumor model. Reprinted with permission [32]. Copyright 2019, Wiley-VCH Verlag GmbH & Co. KGaA, Weinheim

Another promising approach involves the use of nanocarriers to combine different chemotherapeutic agents, thus optimizing the therapeutic efficacy while minimizing additive side effects. For instance, Jiang et al. developed combretastatin A4 nanodrug (CA4-NPs) and matrix metalloproteinase 9 (MMP9)-activated doxorubicin prodrug (MMP9-DOX-NPs) (Fig. 1A) [32]. The sequential delivery of CA4-NPs and MMP9-DOX-NPs increased tumor-selective drug release by amplifying MMP9 expression and enhanced antitumor efficacy with a tumor inhibition rate of 88.2% (Fig. 1B,C). The cooperative strategy resulted in a 1.8-fold increase in efficacy compared with the noncooperative controls (Fig. 1D) [32]. Moreover, Wang et al. first synthesized a glutathione-responsive and pH-responsive cisplatin prodrug (PEG-ADH-DPA-DDP) and then constructed cisplatin prodrug and PTX co-loaded nanoparticles (DDP-P/PTX NPs) [33]. The nanoparticles exhibited redox-sensitive and pH-triggered drug release in a murine model of lung cancer, resulting in high tumor distribution, low systemic toxicity, and synergistic anti-tumor effects [33]. Similarly, Liu et al. engineered lipid-polymer hybrid nanoparticles (LPNs) as a co-delivery system of PTX and triptolide. The combined advantages of both polymeric nanoparticles and liposomes resulted in synergetic antitumor effects with minimal systemic toxicity [34].

### Radiotherapy sensitizer

Radiotherapy is one of the most commonly used therapies for lung cancer patients, regardless of their disease stage [35]. The types of radiation treatment used include systemic, external beam and internal radiotherapies. However, the overall survival rate is still far from ideal for patients treated radiotherapy. This is largely due to the development of radioresistance, which is typically caused by the prevalence of hypoxia and the plasticity of cancer stem cells [36, 37]. Thus, it is of great importance to design radiosensitizers specific for lung cancer radiotherapy applications. Nanomaterials containing elements with high atomic number (Z) have emerged as ideal radiosensitizers. These nanoplatforms can increase the dose of radiation energy absorbed in the tumors and thus improve the therapeutic efficacy of conventional radiotherapy [38]. Nanoparticles based on Au (gold), Bi (bismuth), and Lu (lutetium-177) have been recognized as potential radiosensitizing materials due to their high X-ray absorption and distinct physicochemical properties [39, 40]. For example, Zhuang et al. developed small interfering RNA (siRNA)-Specificity Protein 1 (SP1) loaded AuNPs (AuNPs-si-SP1) [41]. SP1 is a transcription factor overexpressed in lung cancer patients and was predicted to have a binding site with granzyme B. Results showed that the nanoparticles increased the radiosensitivity of lung cancer by reducing cell viability and survival through inhibiting SP1 and upregulating granzyme B [41]. Moreover, Xiao et al. loaded adipose-derived mesenchymal stromal cells with radiosensitive bismuth selenide (Bi_2_Se_3_) NPs (AD-MSCs/Bi_2_Se_3_) to enable targeted radiotherapy of NSCLC [42]. AD-MSCs/Bi_2_Se_3_ could efficiently accumulate at tumor site and further enhance radiotherapeutic efficacy upon X-ray irradiation in orthotopic A549 tumor-bearing mice [42]. In addition, radiotherapy supported by selenium nanoparticles (nano-Se) could induce cell apoptosis and prevent NSCLC proliferation, migration, and invasion [43].

**Fig. 2:**
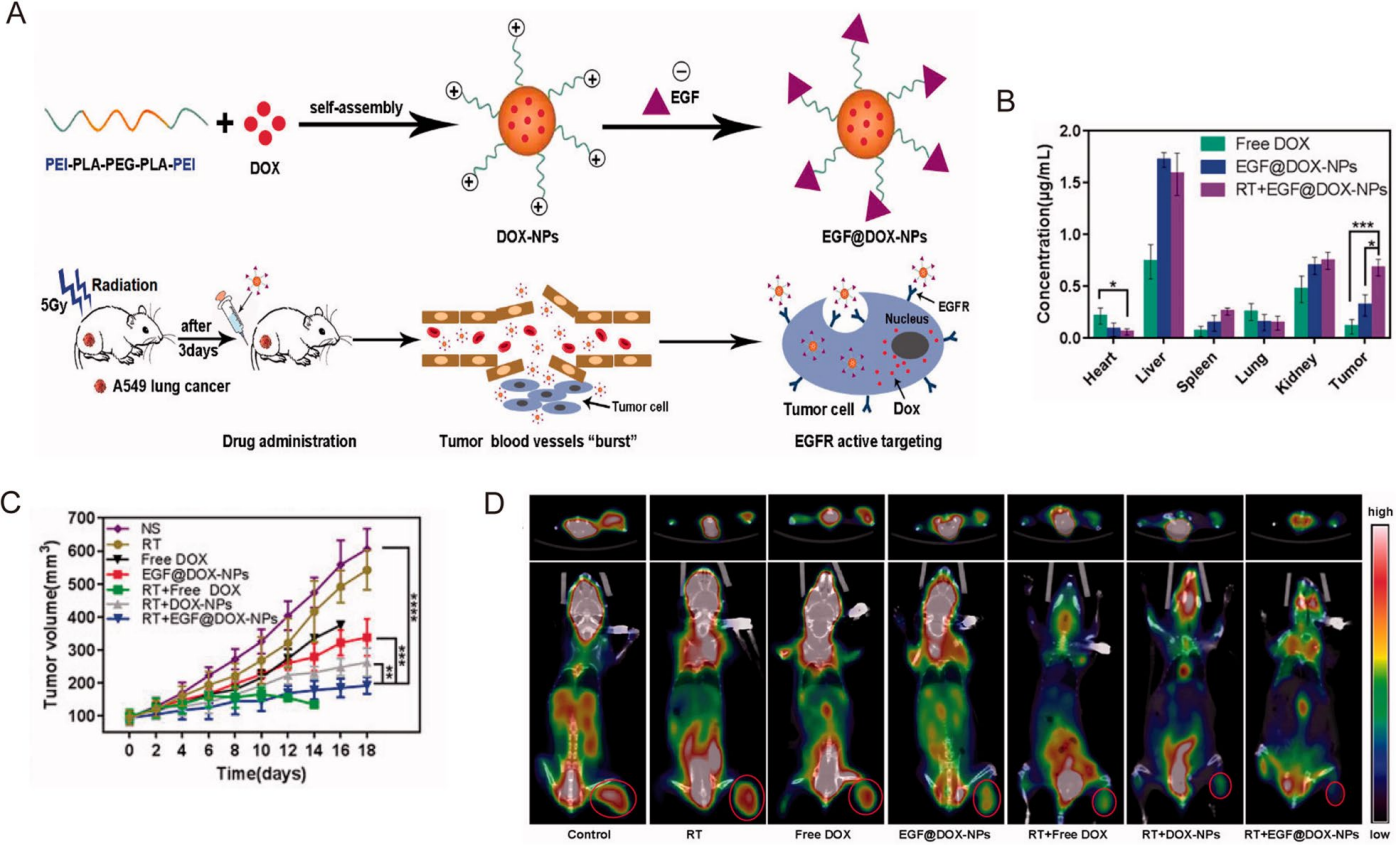
** A** Schematic illustration of the NPs preparation and radiation-induced drug aggregation. **B** DOX concentration in the vital organs. **C** Tumor volume change of different groups in lung tumor xenografts mouse model. **D** Representative micro PET/CT images of resected tumor and mice with different drugs. Reprinted with permission [48]. Copyright 2022, The Author(s)

In clinical practice, radiotherapy is usually combined with other therapies, such as chemotherapy and targeted therapy, to achieve better outcomes for lung cancer patients. Chemoradiotherapy with cisplatin and etoposide is one of the primary treatment methods for both SCLC and NSCLC patients. However, the high interstitial pressure, low blood flow, hypoxia, and acidosis in the TME can limit drug accumulation in the tumor tissues [44, 45], further decreasing the efficacy of radiotherapy. Nanomaterials may be synthesized with relative ease to provide versatility and multifunctionality that can enhance radiotherapy efficacy by alleviating tumor hypoxia or improving cytotoxic effects via reactive oxygen species (ROS) generation [40, 46]. For example, Zhang et al. prepared biocompatible polylactic-co-glycolic acid (PLGA)-PEG polymeric NPs to co-deliver cisplatin and etoposide in a murine model of NSCLC [47]. In conjunction with radiotherapy, the therapeutic efficacy was significantly increased in two murine lung cancer models without inducing additional toxicity [47]. In another study, Wang et al. designed and constructed epidermal growth factor (EGF)-modified doxorubicin nanoparticles (EGF@DOX-NPs) using biocompatible polyethylenimine (PEI) coated polylactic acid (PLA)-PEG-PLA copolymer (Fig. 2A) [48]. They administered a single dose of 5 Gy X-ray radiation to locally burst the tumor vasculature and promote macrophage infiltration. This method significantly increased the accumulation of EGF@DOX-NPs in the tumor tissues (0.68 ± 0.08 µg/mL of RT + EGF@DOX-NPs vs. 0.11 ± 0.07 µg/mL of free DOX) which resulted in superior tumor inhibition effects (Fig. 2B,C) [48]. In addition, in vivo micro PET/CT showed that the combination of radiotherapy with EGF@DOX-NPs significantly inhibited glucose metabolism of tumors (Fig. 2D). Note that the above approach may produce varying tumor permeability and nano-radiosensitization effects on a case-by-case basis due to the heterogeneity and complexity of the tumor extracellular matrix.

### Combination with immunotherapy

#### Immune checkpoint immunotherapy

Immunotherapy, especially immune checkpoint therapy, has been widely studied and applied as treatment for lung cancer in the past few years. Immune checkpoints, which function as negative regulators of immune activation, are proteins on the surface of T cells and other immune cells [49]. The most widely accepted ICIs include monoclonal antibodies targeting CTLA-4 (ipilimumab), PD-1 (nivolumab, pembrolizumab), or PD-L1 (durvalumab, atezolizumab, avelumab). There is sufficient clinical evidence to support the benefits and use of these ICIs in patients with lung cancer [50, 51]. ICIs can also induce a spectrum of immune-related adverse effects (irAEs) as a result of their immunologic mechanisms of action [50]. However, some patients may not respond to ICIs treatment (for example, about 20% of NSCLC patients) [52]. Thus, combination immunotherapy is a promising strategy to effectively increase the sensitivity and availability of ICIs.

It has been found that nanomedicines can significantly improve therapeutic efficacy of ICIs for lung cancer [53]. For instance, Yang et al. successfully synthesized nanomicellar encapsulated-PTX (nano-PTX) that induced immunogenic cell death (ICD) and triggered an immune response in LL/2 lung cancer model [54]. Moreover, nano-PTX upregulated the expression of PD-L1 in immune cells and tumor cells. Thus, the co-treatment with PD-1 antibody could effectively suppress tumor progression and prolong survival [54]. Similarly, Wang et al. constructed cisplatin-NPs by loading cisplatin inside a PLGA-graft-methoxy PEG complex for radiation-induced ICD therapy. As-prepared cisplatin-NPs could amplify radiation and improve ICD efficacy via enhanced CD8^+^ T cells priming and chemokine (C-X-C motif) ligand 10 (CXCL10) secretion [55]. As a result, the combination of cisplatin-NPs, radiotherapy and anti-PD-1 significantly inhibited tumor growth compared to the combination with molecular cisplatin drug in murine models of lung cancer (primary tumor volume on day 14: 173 vs. 653 mm^3^) [55].

**Fig. 3:**
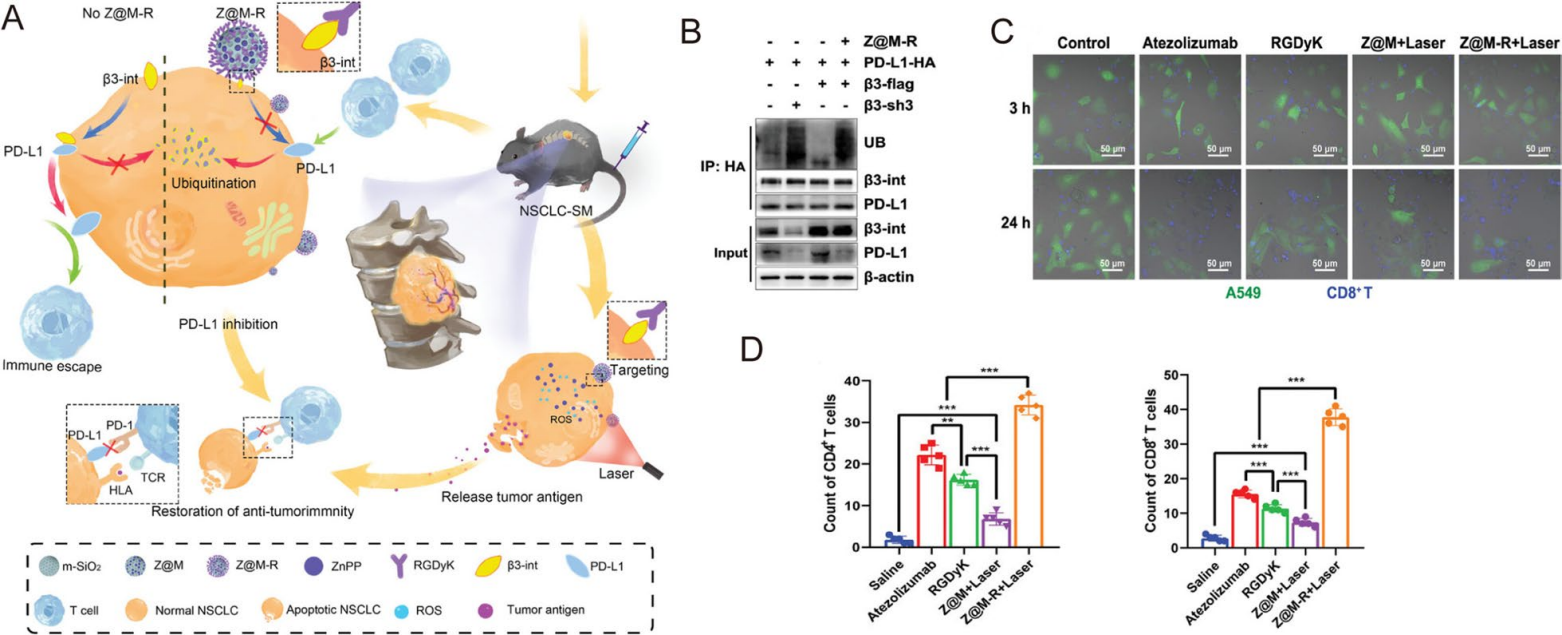
** A** Schematic illustration of the biological mechanism of Z@M-R. **B** Z@M-R promoting PD-L1 ubiquitination in A549 cells. **C** Immunofluorescence images of β3-int-overexpressing A549 cells and activated CD8^+^ T cells co-culture systems. **D** Quantitative analysis of CD4^+^ and CD8^+^ T cells in tumor tissues with different treatments. Reprinted with permission [58]. Copyright 2022, Wiley-VCH GmbH.

In addition to T cells, PD-1 expression has been identified in many other immune cell types, especially tumor-associated macrophages (TAMs) [49]. For example, Xu et al. fabricated nanodiamond-polyglycerol-doxorubicin conjugates (nano-DOX) and found that nano-DOX could induce PD-L1 in NSCLC cells and PD-1 in TAMs through the activation of the HMGB1/RAGE/NF-κB pathway [56]. They showed that nano-DOX and PD-1 blockade could repolarize TAMs into an M1-like phenotype, subsequently triggering synergistic antitumor effect in NSCLC xenografts [56]. In another study, Liu et al. designed gold nanoprisms (GNPs) for both PTT and PD-L1 siRNA delivery. The GNPs-hPD-L1 siRNA sevred as an effective nanoplatform for downregulating PD-L1 expression and photoacoustic imaging as well as photothermal agents in the meantime. Thus, the synergistic therapeutic effects of phototherapy and immunotherapy could be realized in both HCC827 cell line and xenograft model [57]. Zhou et al. found that integrin β3 (β3-int) is highly upregulated in NSCLC patients with spinal metastasis (NSCLC-SM), and the inhibition of β3-int would lead to the ubiquitin degradation of PD-L1. Thus, β3-int serves as a potential target for blockade immunotherapy [58]. In the study, they functionalized mesoporous silicon nanoparticles with Arg-Gly-Asp-d-Tyr-Lys (RGDyK), a β3-int inhibitor, and zinc protoporphyrin (ZnPP) (Z@M-R) (Fig. 3A). Z@M-R showed efficient promotion effect on ubiquitination degradation of PD-L1 in A549 cells (Fig. 3B). Through ZnPP-induced PDT and RGDyK-induced PD-L1 blockade, Z@M-R nanoparticles increased CD8^+^ cytotoxic T-cell proliferation and exhibited significant immunotherapeutic effects owing to the increased infiltration of CD4^+^ and CD8^+^ T cells (more than 30 times compared to saline controls) in the tumor tissues of NSCLC-SM models (Fig. 3C,D) [58].

#### Cancer vaccines

**Fig. 4:**
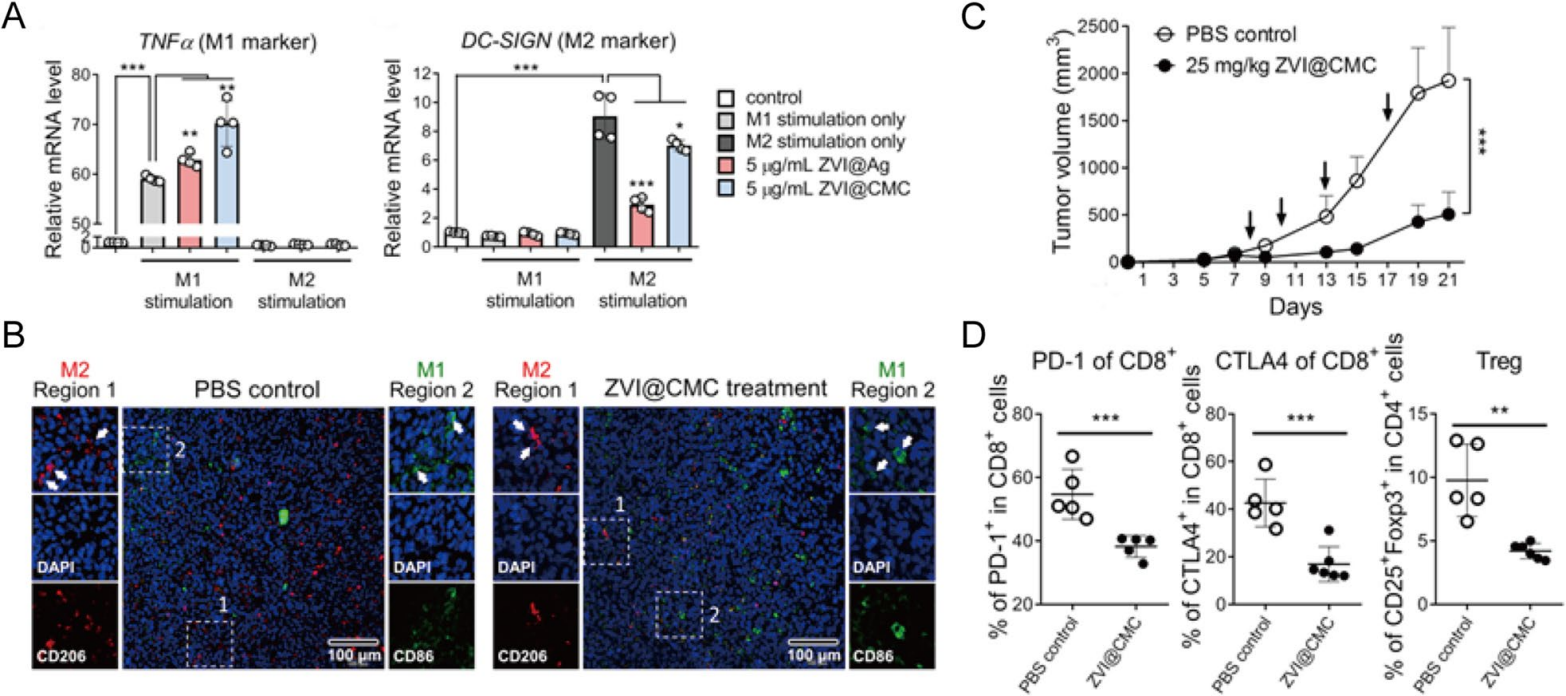
** A** Expressions of TNFα (M1 marker) and DC-SIGN (M2 marker) measured by RT-qPCR in THP-1 macrophages treated with ZVI-NPs. **B** Immunofluorescent staining of macrophages in tumor tissue sections. **C** Tumor volume changes of different groups. **D** The proportions of different T cells in tumors analyzed by flow cytometry analysis. Reprinted with permission [61]. Copyright 2021, The Author(s)

Besides ICIs, a variety of immunotherapies involving various cytokines, agonists, antagonists, antibody-drug conjugates, nucleic acids and so on have been investigated as potential treatments for lung cancer in both preclinical and clinical studies [51]. However, the uncontrolled biodistribution of these small molecules often induces dose-dependent irAEs, such as cytokine-releasing storms, thus hampering their clinical use. On the other hand, cancer vaccines have been designed to prolong the antitumor response without inducing adverse off-target effects [59]. In particular, emerging nanoplatforms have shown to improve the efficacy of cancer immunotherapy via specific TME regulation, sustained antigen release, and enhanced immune stimulation [53, 60]. For example, Hsieh et al. developed zero-valent-iron nanoparticles (ZVI-NPs) that provide immunity against lung cancer by remodeling TAMs to antitumor M1 phenotype both in vitro and in vivo (Fig. 4A, B). When compared with control, ZVI-NPs could inhibite tumor growth by 4 times (Fig. 4C)and enhance lymphocytic immunity by decreasing the proportions of PD-1^+^ cells and CTLA4^+^ cells in tumor-infiltrating CD8^+^ T cells by 20% and reducing the amount of regulatory T cells (Tregs) by half (Fig. 4D) [61].

In another study, Koh et al. designed a novel cancer vaccine based on nanoemulsion (NE) and TLR7/8 agonist (R848) that could convert myeloid-derived suppressor cells (MDSCs) into mature myeloid cells as well as M2 macrophages into M1 macrophages [62]. Notably, this cancer vaccine showed adjuvant therapeutic efficacy toward anti-PD-1 immunotherapy. The combination therapy group exhibited stronger antitumor ability (P = 0.0002) compared with anti-PD-1 monotherapy. The control and anti-PD-1 groups showed tumor progression, while the combination group showed tumor-free survival in 4 out of 9 mice [62]. Similarly, Ye et al. synthesized a TME-modeling neobavaisoflavone nanoemulsion [63]. Such nanoemulsion could induce phenotypic change in macrophages (M2 to M1), increase natural killer (NK) cell number, and reduce the infiltration of immune-suppressive cells (e.g. Tregs and MDSCs). As a result, lung cancer progression was suppressed by nearly 3 times compared to the single neobavaisoflavone treatment group in an A549 lung cancer xenograft model [63].

Nanomedicine formulations can promote cancer vaccine-like effects via multiple stimulations and long-term release. For example, advanced injectable smart hydrogels (ISHs) were employed as a cancer vaccine platform. After administration, controlled degradation of ISHs could ensure persistent release of antigen-loaded nano-sized polyplexes and granulocyte-macrophage colony-stimulating factor (GM-CSF), thereby effectively suppressing human lung carcinoma in vivo [64]. Similarly, Oh et al. utilized gelatin-based hydrogel to co-deliver DCs, oncolytic adenovirus co-expressing interleukin (IL)-12, and GM-CSF, which resulted in sustained, synergistic cancer vaccine therapeutic effects [65].

### Ablative therapy optimization

#### Radiofrequency ablation (RFA)

RFA is a one-step, minimally invasive procedure that is well tolerated in medically inoperable patients. RFA guided by computed tomography (CT) is feasible for treating early lung cancer as well as pulmonary metastases from a wide range of primary tumors with limited lung tissue damages [66, 67]. Nonetheless, the efficacy of RFA is limited due to the risk of incomplete ablation, so additional intervention is often required. Local RFA treatment combined with systemic therapy, such as chemotherapy, targeted therapy, and immunotherapy, has been demonstrated to increase the survival rate of lung cancer patients by reducing lung cancer recurrence [68]. Moreover, it has been recognized that RFA can potentially trigger specific immune response via tumor antigen release. For instance, Xu et al. suggested that intratumoral administration of CpG (TLR9 agonists) followed by RFA could enhance RFA-induced cytotoxic T lymphocyte (CTL) responses. This combined therapy halted the growth of primary RFA-treated and distant untreated tumor as well as the spread of lung metastasis [69]. Interestingly, Li et al. showed that local RFA combined with melatonin (MLT) could significantly improve the clinical outcomes of early lung cancer patients with multiple pulmonary nodules [70]. Local RFA treatment first initiated the recruitment of NK cells to the tumor site, and MLT further promoted the antitumor immune response from RFA-induced NK cells recruitment, thus exerting synergistic inhibitory effects on lung tumor growth [70]. Based on these findings, the combination of nanomedicine and RFA may thus give way to improved antitumor immune response. For example, Yang et al. co-encapsulated lipoxidase and hemin (an iron catalyst) in PLGA using a CaCO_3_-assisted double emulsion method (HLCaP) [71]. With RFA, the HLCaP nanoreactors induced effective lipid peroxidation that resulted in immunogenic cell death. This treatment further combined with anti-PD-1 immunotherapy resulted in growth inhibition of residual tumors, thus preventing tumor recurrence and lung metastasis [71].

#### Phototherapy

Phototherapy is a non-invasive procedure that serves as an alternative treatment for patients with localized central NSCLC who are unable to undergo surgical resection [16]. Phototherapy, including photothermal and photodynamic therapy (PTT and PDT), uses laser irradiation to realize therapeutic effects. Upon near-infrared (NIR) laser irradiation, PTT achieves localized heating to ablate tumor tissues [72], while PDT generates free radicals and ROS to induce cellular damages [73]. However, it is challenging to achieve desirable phototherapeutic effects with conventional small molecular agents due to their non-specificity, low photoconversion efficiency, and poor bioavailability. On the other hand, nanomaterials can be modified to carry photothermal agents (PTA) or photosensitizers, and their designs can be tuned to encourage tumor accumulation. To date, a broad array of nanoprobes have been studied as PTT and PDT agents, including magnetic nanoparticles, metal nanoparticles, carbon nanotubes, two-dimensional (2D) materials, metal-organic frameworks (MOFs), quantum dots, NIR dyes encapsulated nanoparticles, and semiconducting polymer nanoparticles [74–76]. For instance, black phosphorus nanosheets (BP NSs), a 2D nanomaterial with high surface area and negative charge, are highly biocompatible with the ability to generate heat and ROS upon laser irradiation, demonstrating the feasibility of using this agent for both PTT and PDT [77].

**Fig. 5:**
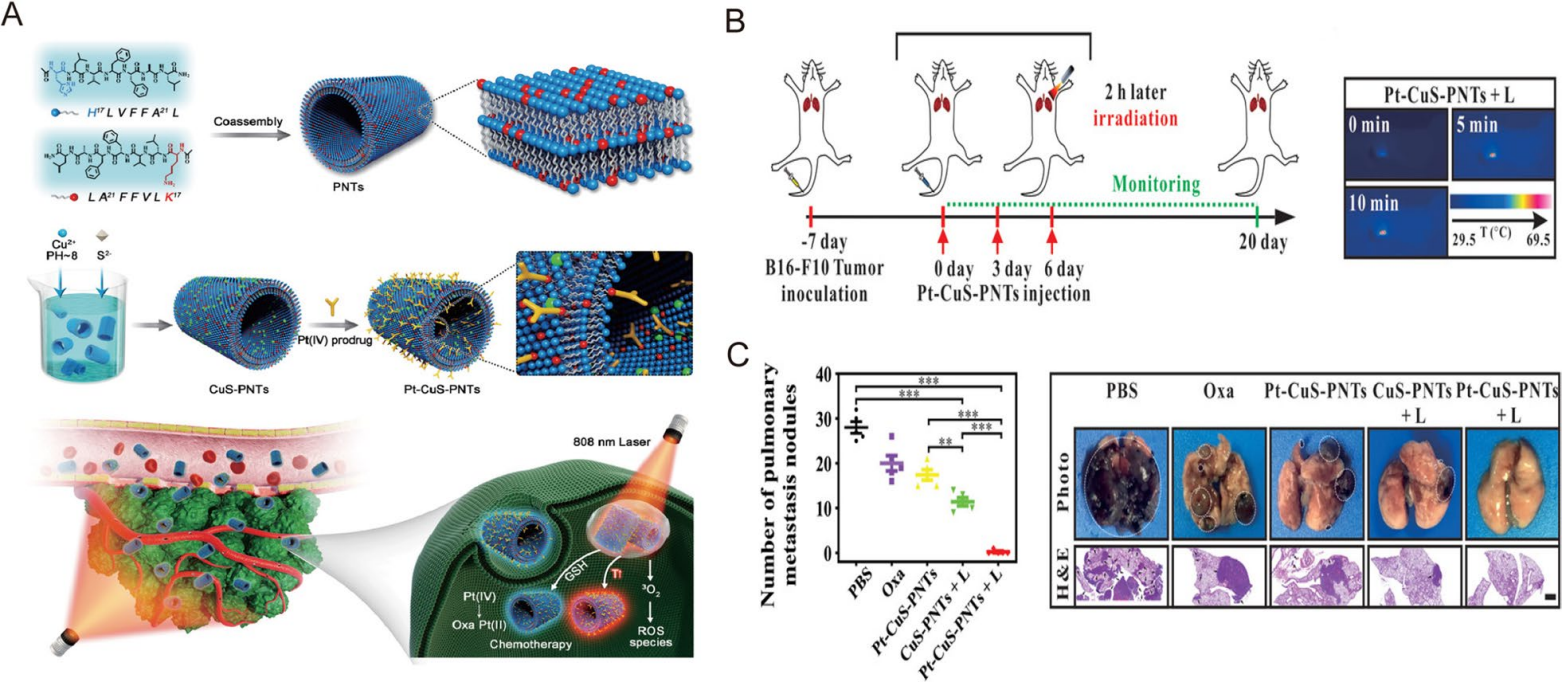
** A** Schematic illustration of Pt–CuS–PNT preparation and the combination cancer therapy. **B** Experimental scheme and infrared thermal images of the combination treatment of lung metastatic melanoma mice model. **C** Quantitative analysis of the lung metastatic nodules and representative pictures and H&E of the lung tissues. Reprinted with permission [80]. Copyright 2019, Wiley-VCH Verlag GmbH & Co. KGaA, Weinheim

Nonetheless, it is well accepted that the complete elimination of tumor tissues is not possible with phototherapy alone, and this may cause tumor recurrence due to the presence of residual tumor cells. Thus, considerable research efforts have focused on combining phototherapy with other types of therapy, such as chemotherapy and immunotherapy, while incorporating nanomedicine to further mediate and optimize the therapeutic performance [78]. For example, Zhang et al. developed a novel nanomedicine formulation by loading BiOI@CuS nanoparticles with doxorubicin, aspirin phenacetin, and caffeine (APC) [79]. When subjected to 980 nm laser irradiation, this nanosystem triggered the release of DOX and APC via photothermal heating, thereby improving the therapeutic outcome while minimizing adverse side effects [79]. To construct a versatile nanoplatform, Lai et al. used peptide nanotubes to biomineralize Cu_2 − x_S nanoparticles and integrated them with an oxaliplatin prodrug (Pt-CuS-PNTs) via covalent interactions (Fig. 5A) [80]. Upon 808 nm laser illumination, this nanoplatform resulted in significant tumor hyperthermia and ROS generation, which contributed to the inhibition of tumor growth and lung metastasis in a B16-F10 melanoma model (Fig. 5B, C) [80].

The immune activating capability of phototherapy has prompted the use of various nanomaterials as cancer vaccines via photo-immunotherapy. For example, Liu et al. developed a novel cancer therapeutic vaccine by encapsulating black phosphorus quantum dots within exosomes and applied the nano-vaccine in a murine subcutaneous lung cancer model [81]. This vaccine demonstrated excellent PTT efficacy through activated host immunity that subsequently increased the number of tumor-infiltrating T-cells [81]. In another study, Huang et al. designed an in situ photothermal nano-vaccine by co-encapsulating immune adjuvant CpG-loaded BP-Au nanosheets with an indoleamine 2,3-dioxygenase inhibitor (NLG919) [82]. The resultant tumor vaccine activated the effector and memory T cells, subsequently suppressing Tregs. This immune response led to remarkable therapeutic effects on both primary and lung metastatic tumors [82].

## Adjuvant nanomedicine for overcoming drug resistance

Drug resistance is a major challenge that causes therapeutic failures and arises as part of any cancer treatment regimens, especially with chemotherapy and molecularly targeted therapy. This problem also occurs with nanomedicine treatments after being applied for some time [83]. Multidrug resistance emerges in lung cancer cells through different intrinsic and acquired mechanisms, including altered cellular targets or driver genes, decreased cellular drug concentrations, altered proliferation, reduced susceptibility to apoptosis, acquisition of epithelial-mesenchymal transition and cancer stem cell-like phenotypes, epigenetic modulation, and tumor heterogeneity [84, 85]. The interactions with tumor stroma and TME also contribute to the development of drug resistance.

Mitochondrial-targeting via nanosystem is a promising strategy for overcoming drug resistance in lung cancer therapy. For instance, Li et al. modified liposomes with dequalinium polyethylene glycol-distearoylphosphatidylethanolamine (DQA-PEG_2000_-DSPE) for mitochondrial-targeting and loaded the liposomes with an anthracycline drug epirubicin or lonidamine [86]. These liposomes induced significant anticancer effects toward drug-resistant A549cDDP lung cancer cells and drug-resistant A549cDDP xenografted BALB/c nude mice [86]. In addition, pH-responsive liposomes modified with KLA peptides allowed for mitochondrial-oriented delivery of PTX, the positively charged KLA peptide could target mitochondria and promote cellular uptake, which further improved the chemotherapeutic efficacy against A549 cells and PTX-resistant A549 cells both in vitro and in vivo, with a tumor growth inhibition of 86.7% [87].

Remodeling or targeting TME with nanomaterials also shows great potency in reversing drug resistance. For example, Tie et al. constructed a folate-modified liposome (F-PLP) for the delivery of a BIM-S plasmid to targeted cancer cells and FRβ-positive macrophages in the TME [88]. BIM-S is an isoform of BCL-2-interacting mediator of cell death (BIM), which is crucial for cell apoptosis following effective targeted therapy, but deficiency in BIM expression usually leads to targeted therapy resistance [88]. Zhang et al. designed a chitosan-coated selenium/cisplatin (CSP) nanoparticle that reduces ROS generation in hypoxic TME and thus avoids HIF-1 activation [89]. Furthermore, CSP nanoparticles could downregulate the proteins responsible for cisplatin resistance, such as glutamate-cysteine ligase modifier subunit and P-glycoprotein. These proteins usually well correlate with the HIF-1α level. CSP nanoparticles exhibited enhanced antitumor efficacy to cisplatin-resistant A549/DDP lung cancer cell lines and xenografts [89].

**Fig. 6:**
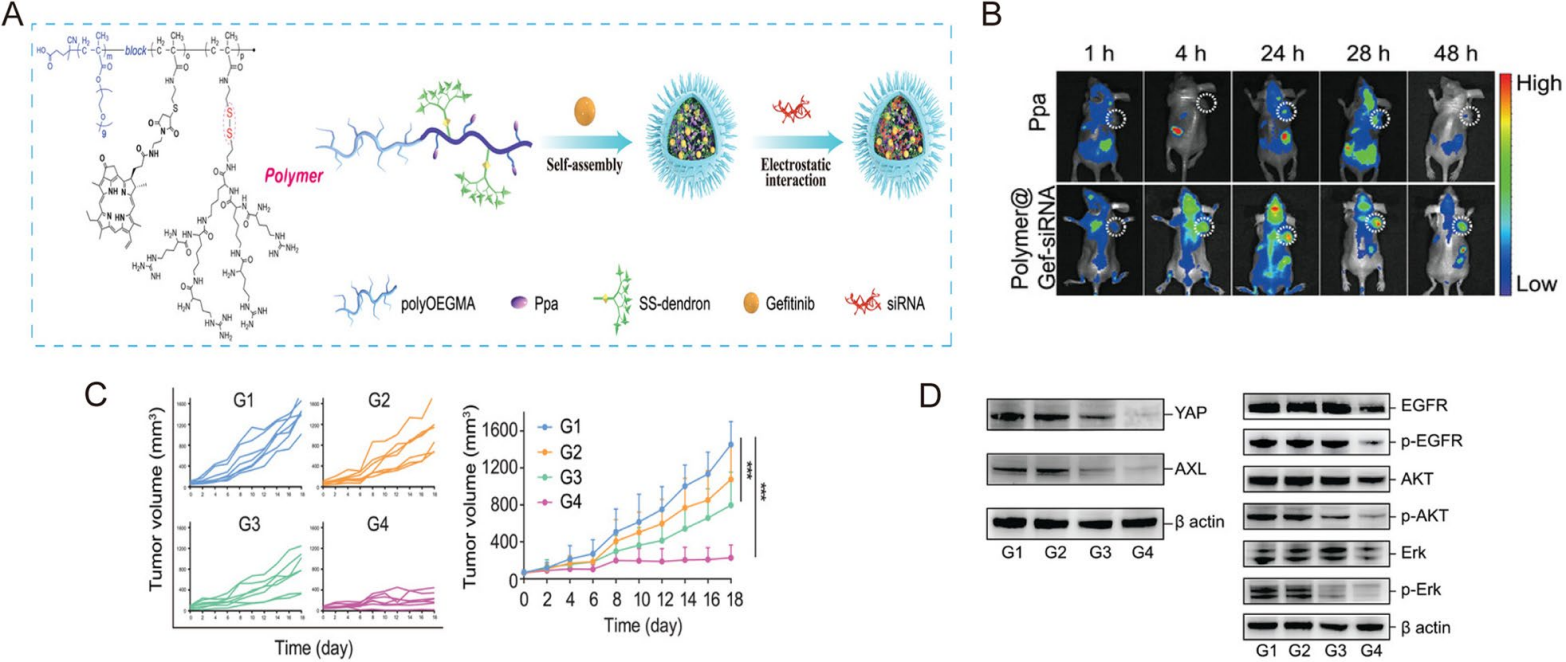
** A** Schematic illustration of the formation of Polymer@Gef-siRNA NPs. **B** Representative in vivo images of PDX-bearing mice. **C** Tumor volume changes of mice treated with saline (G1), free Gefitinib (G2), Polymer@Gef-YAP-siRNA (G3), and Polymer@Gef-YAP-siRNA + Laser (G4), respectively. **D** Western blotting images of tumor tissues. Reprinted with permission [90]. Copyright 2022, Wiley-VCH GmbH.

Targeting the critical proteins in TKI resistance or genetic mutations via nanomaterial-based nucleic acids delivery has emerged as another promising strategy [90, 91]. For instance, Huang et al. synthesized a nano-cocktail composed of amphiphilic and block-dendritic-polymer-based nanoparticles (NPs) for targeted co-delivery of EGFR-TKI gefitinib and YAP-siRNA (Fig. 6A) [90]. Compared to Ppa, this nano-cocktail showed strong accumulation in tumors after intravenous injection in gefitinib-resistant NSCLC patient-derived xenografts (PDXs) (Fig. 6B). Moreover, the tumor growth was significantly inhibited with a tumor growth inhibition rate of 86.7% and the expression level of YAP, AXL, EGFR, AKT, and ERK in the tumor tissue was decreased by Polymer@Gef-YAP-siRNA with laser (Fig. 6C,D) [90]. Overall, exploring the underlying pathological processes and engineering relevant, advanced nanomedicines may give way to fighting drug resistance in lung cancer.

## Conclusion and future perspectives

As illustrated above, nanomedicine-based lung cancer therapy has shown great potential in improving conventional therapies with higher efficacy and lower systemic adverse events. Currently, there is a broad array of novel therapeutic formulations under investigation, each with distinct characteristics suited for different lung cancer treatments. Some are already approved for clinical use, while others are being studied in preclinical or early-stage clinical trials. Despite the increasing presence of nanomedicine in adjuvant lung cancer therapies, there are several challenges that hinder their practicality and utility. Factors like loading efficiency, mass production, biodistribution, pharmacokinetics and toxicity still need to be resolved or optimized.

The clinically approved nanomedicines for lung cancer are mostly delivered via systemic administration. Non-specific distribution and off-target effects due to the lack of active targeting have limited the efficacy of nanomedicines. Hence, to address these overarching barriers, versatile, targeted strategies have emerged to warrant efficient adjuvant treatments [92]. For example, surface modifications using ligands that target tumor cell receptors could facilitate site-specific delivery [93]. In lung cancer, the prominent cell surface receptors include folate, EGFR, α_v_β_3_ integrin, CXCR4 and so forth [92]. Furthermore, biomimetic nanoparticles composed of naturally-derived biomembranes, such as extracellular vehicles and extracted plasma membranes, have shown great advantages in improving cancer treatment in the preclinical setting [29, 94]. The biomimetic nanocarriers may exert different functions, such as immune escape, homologous targeting, and long circulation, when encountering different source cell types, such as immune cells, tumor cells, red blood cells, and platelets [94]. These tumor-targeting strategies using nanomedicines have also greatly reduced systemic toxicity, thus ensuring better safety profiles for future clinical lung cancer treatments.

Nanomaterials can be designed to respond to specific stimuli in the TME, such as acidic pH, enzymes, hypoxia, ROS and glutathione levels [45]. Stimuli-responsive materials can achieve efficient and site-specific delivery as well as control the release of therapeutic payloads [45, 92]. These newly developed nanoformulations have proven to be beneficial and have the potential to advance the field of nanomedicine-based lung cancer therapy. Thus, combining the characteristics of TME stimuli-responsive nanoparticles and biomimetic ones is of great significance. According to its natural biocompatibility, biomimetic nanomedicines should have better translational prospects for future clinical applications in lung cancer treatments. If combined with some TME stimuli-responsive nanomaterials, the targeting effect can be achieved in the whole therapeutic process, thus providing a promising strategy for cancer therapies with lower doses and less systemic effects. However, the modification and loading process may alter some original properties of those biomimetic nanocarriers, and the inner mechanisms of transportation in vivo have not been fully elucidated. Therefore, since the preparation methods may be different from lab to lab, the stability and toxicity of these nanomedicines still require more exploration, and some standard procedures are also necessary. Anyhow, nanomedicine-based adjuvant therapy has demonstrated strong enhancement of conventional lung cancer therapies, and new therapeutic regimens have emerged, due to research around adjuvant nanomedicines. For instance, as discussed above, photo-immunotherapy using nanovaccines may provide promising solutions for treating lung cancer.

Overall, nanomedicines have offered us new insights into lung cancer theranostics. The field has seen tremendous progress in the last few years, and the advances have greatly improved the therapeutic outcomes and safety of conventional therapies. Given recent developments in this research area, growing interest in using adjuvant nanomedicines for lung cancer treatments is highly anticipated.

## Data Availability

Not applicable.

## Abbreviations

NSCLC: Non-small cell lung cancer
SCLC: Small cell lung cancer
EPR: Enhanced permeability and retention
RFA: Radiofrequency ablation
PDT: Photodynamic therapy
PTT: Photothermal therapy
NGS: Next-generation sequencing
TKIs: Tyrosine kinase inhibitors
EGFR: Epidermal growth factor receptor
KRAS: Kirsten rats arcomaviral oncogene homolog
MET: Mesenchymal epithelial transition factor
NTRK: NeuroTrophin Receptor Kinase
RET: Ret proto-oncogene
ICIs: Immune checkpoint inhibitors
TLR: Toll-like receptor
PTX: Paclitaxel
PEG: Polyethylene glycol
TME: Tumor microenvironment
CA4: Combretastatin A4
MMP9: Matrix metalloproteinase 9
DOX: Doxorubicin
siRNA: Small interfering RNA
SP1: Specificity protein 1
AD-MSCs: Adipose-derived mesenchymal stromal cells
ROS: Reactive oxygen species
PLGA: Polylactic-co-glycolic acid
EGF: Epidermal growth factor
PEI: Polyethylenimine
irAEs: Immune-related adverse effects
ICD: Immunogenic cell death
CXCL: Chemokine (C-X-C motif) ligand
TAMs: Tumor-associated macrophages
GNPs: Gold nanoprisms
MDSCs: Myeloid-derived suppressor cells
Tregs: Regulatory T cells
NE: Nanoemulsion
ISHs: Injectable smart hydrogels
GM-CSFs: Granulocyte-macrophage colony-stimulating factor
IL: Interleukin
CTL: Cytotoxic T lymphocyte
MLT: Melatonin
NIR: Near-infrared
PTA: Photothermal agents
MOFs: Metal-organic frameworks
BP: Black phosphorus
BIM: BCL-2-interacting mediator
CSPs: Chitosan-coated selenium/cisplatin
PDXs: Patient-derived xenografts
